# Perceived barriers to the uptake of health services among first-year university students in Johannesburg, South Africa

**DOI:** 10.1371/journal.pone.0245427

**Published:** 2021-01-22

**Authors:** Nozipho Orykah Musakwa, Jacob Bor, Cornelius Nattey, Elisabet Lönnermark, Peter Nyasulu, Lawrence Long, Denise Evans

**Affiliations:** 1 Health Economics and Epidemiology Research Office, School of Clinical Medicine, Faculty of Health Sciences, University of the Witwatersrand, Johannesburg, South Africa; 2 Department of Global Health, Boston University School of Public Health, Boston University, Boston, MA, United States of America; 3 Department of Epidemiology, Boston University School of Public Health, Boston University, Boston, MA, United States of America; 4 Department of Infectious Diseases, Sahlgrenska University Hospital, Gothenburg, Sweden; 5 Division of Epidemiology & Biostatistics, Faculty of Health Medicine & Health Sciences, Stellenbosch University, Cape Town, South Africa; 6 Division of Epidemiology & Biostatistics, School of Public Health, Faculty of Health Sciences, University of the Witwatersrand, Johannesburg, South Africa; Fred Hutchinson Cancer Research Center, UNITED STATES

## Abstract

**Background:**

Young people face many barriers to accessing appropriate health care services including screening for HIV and tuberculosis (TB). The study aimed to identify perceived barriers to the uptake of health services among young adults entering the tertiary education system in South Africa.

**Methods:**

We conducted a cross-sectional study among first-year students aged 18–25 years, registered at one of three universities in Johannesburg, South Africa, in 2017. Participants completed a self-administered paper-based questionnaire. We describe perceived barriers to accessing health services, stratified by gender and recent engagement in TB or HIV services, together with sources of information about HIV and TB.

**Results:**

Seven hundred and ninety-two (792) students were included in the study of which 54.8% were female. Perceived barriers to accessing services included long waiting time (n = 342,43.2%), attitude of health workers (n = 263,33.2%), lack of sufficient information/poor health literacy (n = 148,18.7%), and inability to leave/stay away from studies (n = 137,17.3%). Among participants who tested for HIV in the past 6 months (n = 400, 50.5%), waiting time and attitude of health care workers were perceived as barriers to accessing services. Compared to males, females were more likely to view attitudes of health workers (40.3% vs. 25.0%; p = 0.001) and inability to leave/stay away from studies (20.5% vs.13.4%; p = 0.025) as potential barriers. While just over half of the students (50.5%; 400/792) in this study had accessed health services in the past 6 months, very few (15.0%) opted to use campus health services, and even less (5%) reported receiving information about HIV and TB from the university itself.

**Conclusion:**

Despite perceived barriers to accessing HIV and TB services off campus, fewer than one in five students starting out at university opted to use campus health services. Campus health services could address many of the barriers unique to university students.

## Introduction

In South Africa, the 15-24-year age group are at an increased risk of HIV infection [1]. In 2017, HIV incidence for young adults between the ages of 15–24 was 1.0% (95% CI 0.86–1.15), translating to an estimated 88 400 new infections [2, 3]. In the same year, WHO and other UN partners launched the Accelerated Action for the Health of Adolescents (AA-HA!), which called for the systematic inclusion of adolescents’ expectations and perspectives in health planning processes [4]. However, many stakeholders have shown inadequate insight into the factors that influence adolescent health. Understanding the specific health needs of young adults could help countries tailor policies to address these specific needs and improve adolescent health [4].

The entrance to comprehensive, quality health care is essential for health promotion and maintenance as well as disease prevention and management [5]. Research has shown that adolescents (10–19 years) and young adults (20–24 years) face challenges utilizing appropriate health care services, including for HIV and tuberculosis (TB) [6, 7]. Adolescents and young adults face difficult and often confusing emotional and social pressures as they move from childhood to adulthood, and therefore the perceived barriers to the uptake of health services are different than those reported by adults [8].

Potential barriers have been classified into individual (patient), provider, and system barriers [7]. Current literature on health care utilization among adolescents and young adults in urban Johannesburg, South Africa cites several barriers to access including: long traveling distance to the clinic, the possibility that attendance at the clinic would be noticed by friends or members of the school, having an elderly caregiver, high transport costs, and long queues at the clinic [9, 10]. Lack of youth-friendly training among staff, lack of a dedicated space for young people [11], waiting times due to inconvenient operating times and low numbers of staff [12–14], poor staff attitudes [15], and stigma [16] have also been commonly identified as perceived barriers for adolescents and young adults accessing health care. More generally, demographic factors that have been associated with access to care include socio-economic status, race, insurance status, and urban-rural location with black Africans, poor, uninsured and rural respondents, experiencing greatest barriers [17].

Each year over 1 million students enroll in universities across South Africa [8]. Twenty percent of these are first-time students who have to cope with the social complexities, complex social networks or unequal power dynamics of this new environment [18]. Little is known about care-seeking patterns of university students and the role of campus health services in providing comprehensive health services for young people. The university campus represents a mode of delivery of youth-friendly services tailored to meeting the healthcare needs of young people [8], and therefore presents an important opportunity to assess health-seeking behavior in this population and identify opportunities to intervene with health promotion activities [8].

This study aimed to understand perceived barriers to the uptake of general health services, and then more specifically the uptake of HIV testing and TB screening services, among first-year university students in Johannesburg, South Africa to demonstrate the challenges that HIV positive students might have in accessing care. Because we know from other work that there are differences between men and women in terms of accessing services; women are more likely to seek and use health services compared to men; men have lower levels of health literacy than women; and men are less likely than women to acknowledge illness or to seek help when sick [19–22], we stratified our results by gender in order to assess differences in health seeking behavior among young men and women. Finally, in order to inform future informational interventions to improve the uptake of services for HIV and TB, we asked students where they received information about HIV and TB from, and where they would prefer to get this information from.

## Methods

### Design

This was a cross-sectional study among first-year university students, aged 18–25, registered between February 2017 and November 2017 at one of the three study sites (two public and one private university) in Johannesburg, South Africa.

### Population and procedures

The universities selected for this study were located in Johannesburg, South Africa. Findings of this study are part of a larger study that was conducted to explore the knowledge and risk perceptions of TB and HIV among high school leavers entering tertiary education [8]. Detailed methods have been reported elsewhere [8]. At the time of the study the universities selected to participate in the study offered primary healthcare, which included sexual and reproductive health, health promotion, nutrition and mental health services but could not offer antiretroviral therapy (ART) to students and rather referred students to other sites that provided ART. Two of the campus clinics charged a fee for services, while the third offered services (e.g., HIV testing, sexual and reproductive health care including family planning and consultations with medication) for free. For the two that charged a fee, the fee varied by university and ranged from a nominal fee (e.g. ZAR20 for consultation with a nurse) to charging full medical aid rates for consultations and medication.

A convenient sample was obtained by approaching first-year students in common areas on the days that study staff visited the university campuses (e.g., library, canteen, lunch area). Students who had completed secondary school more than three years ago and those who has been a university student for more than one year (e.g., those completing a bridging year prior to registering for a formal degree) were excluded.

Study staff approached potential participants and those who met the initial pre-screening criteria (e.g., first year student, 18 years of age and older and registered at the university) were invited to participate. Thereafter, study staff provided a detailed explanation of the study, confirmed eligibility and eligible students were asked to provide written informed consent. Students enrolled in the study completed a self-administered, paper-based, structured questionnaire, in English, the primary language of instruction at all three universities. Details on the questionnaire and the sample size are described in the original paper [8].

Data were collected on paper questionnaires and entered into REDCap, an electronic data capture tool, hosted by the University of the Witwatersrand [23]. Data were then exported into STATA Version 14 (StataCorp, Texas, USA) for further processing and analysis. Fictitious records (i.e., where students fabricated data) were manually identified through pre-specified quality-control procedures and were removed prior to the analysis.

### Study variables

Data were collected on demographics, socio-economic status, HIV status, and HIV or TB testing history. Socio-economic status (SES) was estimated using an asset index, based on ownership of assets, power source, and food security quality [24, 25]. Based on this index, SES of households was divided into three categories (i.e., low, medium and high) representing proxies for SES. The methods and data used to derive this variable are described in the original paper [8].

We collected data on general health-seeking behavior such as; where students would go to access services, when the last time students went to the health center/clinic, the reason for going, and the mode of transport they would use to get there. To understand perceived barriers, we asked students what they would have to do to visit the health care facility. We considered both the financial and opportunity costs (e.g., time away from studies) to the individual as potential barriers. To assess individual and facility-related barriers for TB/HIV, students were asked to identify perceived barriers that would prevent them from seeking health care for TB/HIV at their nearest health center. All questions relating to perceived barriers were derived from published questionnaires [26–28]. Lastly, students were asked where they received information about HIV and TB from, and where they would prefer to get this information from (S1 Table).

### Statistical analysis

First, to assess existing care seeking patterns, we describe the most recent engagement in general health services and the reasons for visiting a health care facility. We also describe recent engagement in HIV testing and TB screening services and choice of service provider (e.g., general practitioner, campus clinic, public hospital or health care center etc.).

Second, to assess perceived barriers to care-seeking, we describe the most common perceived individual and facility-related barriers to the uptake of for TB and HIV services and also report what respondents believed they would need in order to visit the clinic or health care facility.

Third, to access whether these barriers differed by gender and past care-seeking experiences, we first report the gender differences in characteristics of students enrolled in the study, the perception of barriers stratified by gender and recent engagement in TB or HIV services (i.e., screened/tested in the past six months) and compare these using the Chi-square test for proportions. Then, we determine the association between gender (female vs. male) or recent engagement in TB or HIV services (i.e., screened/tested in the past six months) and perceived barriers to the uptake of health services. To do this we used a log-binomial regression model to estimate the crude Relative Risk (RR) with the corresponding 95% confidence interval.

Fourth, to assess student preferences for sources of information about HIV and TB, we graphically present sources where students report receiving information and where they would prefer to get information about HIV and TB from.

This study was approved by the Human Research Ethics Committee (Medical) of the University of the Witwatersrand (Wits HREC M161019). Each university provided permission to recruit on campus and all participants provided written informed consent to participate in the study.

## Results

A total of 811 students, 89% of those screened, were enrolled in the study. After fictitious data (n = 5), duplicates (n = 11), and those with incomplete consent (n = 3) were excluded, 792 students were included in the analysis (Table 1). A flow diagram summarizing participant enrolled can be found in the original paper [8].

**Table 1:** Characteristics of the students enrolled in the study (n = 792).

Characteristic		All N = 792 (%)	Female* N = 434 (54.8%)	Male* N = 352 (44.4%)
Age, years	18–19	254 (32.1%)	162 (37.3%)	92 (26.1%)
	20–25	521 (65.8%)	265 (61.1%)	252 (71.6%)
	Missing	17 (2.1%)	7 (1.6%)	8 (2.3%)
Nationality	Non-South African	120 (15.2%)	68 (15.7%)	52 (14.8%)
	South African	584 (73.7%)	319 (73.5%)	260 (73.5%)
	Missing	88 (11.1%)	47 (10.8%)	40 (11.4%)
Ethnicity	Black	722 (91.2%)	397 (91.2%)	321 (91.2%)
	White	27 (3.4%)	16 (3.7%)	10 (2.8%)
	Coloured	20 (2.5%)	12 (2.7%)	8 (2.3%)
	Indian	17 (2.1%)	7 (1.6%)	10 (2.8%)
	Missing	6 (0.8%)	2 (0.8%)	3 (0.9%)
Health insurance type	Private health insurance	256 (32.3%)	109 (31.0%)	145 (33.4%)
	None	461 (58.2%)	213 (60.5%)	246 (56.7%)
	Other	3 (0.4%)	0 (0.0%)	3 (0.7%)
	Missing	72 (9.1%)	30 (8.5%)	40 (9.2%)
Socio-economic status	Low	207 (26.1%)	114 (26.3%)	92 (26.1%)
	Medium	223 (28.2%)	130 (30.0%)	92 (26.1%)
	High	190 (24.0%)	103 (23.7%)	84 (23.9%)
	Missing	172 (21.7%)	87 (20.0%)	84 (23.9%)
Type of tertiary institution	Private	84 (10.6%)	52 (12.0%)	29 (8.2%)
	Government subsidized	708 (89.4%)	382 (88.0%)	323 (91.8%)
Faculty	Science and Engineering (including Information Technology)	277 (35.0%)	119 (27.4%)	157 (44.6%)
	Humanities and Education	367 (46.3%)	235 (54.2%)	128 (36.4%)
	Health	43 (5.4%)	24 (5.5%)	19 (5.4%)
	Missing	105 (13.3%)	56 (12.9%)	48 (13.6%)
**HIV and TB**				
Ever had an HIV test	Yes	416 (52.5%)	230 (53.0%)	184 (52.3%)
	In the past 6 months	400/416 (96.2%)	230 (100%)	168 (91.3%)
	Never tested	242 (30.6%)	136 (31.3%)	103 (29.3%)
	Refused to answer/missing	134 (16.9%)	68 (15.7%)	65 (18.4%)
Frequency of testing	More than 5 years ago	3/416 (0.7%)	3/230 (1.30%)	0/184 (0.0%)
	Once in the last 5 years	127/416 (30.6%)	67/230 (29.2%)	60/184 (17.1%)
	Twice in the last 5 years	107/416 (25.7%)	53/230 (23.0%)	53/184 (15.1%)
	Three time in the last 5 years	60/416 (14.4%)	32/230 (13.9%)	28/184 (8.0%)
	>4 times in the last 5 years	119/416 (28.6%)	75/230 (32.6%)	43/184 (12.2%)
HIV status	Positive	24 (3.0%)	15 (3.5%)	9 (2.6%)
	On ART	15/24 (62.5%)	6/15 (40.0%)	9/9 (100.0%)
	Negative	559 (70.6%)	304 (70.1%)	251 (71.3%)
	Don’t know	153 (19.3%)	86 (19.8%)	66 (18.8%)
	Refuse to answer/missing	56 (7.1%)	29 (6.7%)	26 (7.4%)
Screened for TB in the past 6 months	No	644 (81.3%)	362 (83.4%)	277 (78.4%)
	Yes	112 (14.1%)	54 (12.4%)	58 (16.5%)
	Refuse to answer	14 (1.8%)	5 (1.2%)	9 (2.6%)
	Don’t know/missing	22 (2.8%)	13 (3.0%)	8 (2.6%)
**Engagement in health services**				
Visited health facility in the last 6 months	Yes	400 (50.5%)	249 (57.4%)	150 (42.6%)
	No	392 (49.5%)	185 (42.6%)	202 (57.4%)
Reason for visit to facility in the last 6 months	Sick	178 (44.5%)	99 (39.8%)	79 (52.7%)
	HIV testing	116 (29.0%)	71 (28.5%)	44 (29.3%)
	Collect medication	15 (3.7%)	10 (4.0%)	5 (3.3%)
	Family planning	48 (12.0%)	44 (17.7%)	4 (2.7%)
	Other	43 (10.8%)	25 (10.0%)	18 (12.0%)
Ever reported visiting a health facility	Yes	728 (91.9%)	406 (93.5%)	318 (90.3%)
	No	64 (8.1%)	28 (6.5%)	34 (9.7%)
Preference for	Public hospital or health care center	387 (48.9%)	192 (44.3%)	194 (55.1%)
	Private doctor or clinic	231 (29.2%)	126 (29.0%)	101 (28.7%)
	Campus clinic	119 (15.0%)	80 (18.4%)	39 (11.1%)
	Other	55 (6.9%)	36 (8.3%)	18 (5.1%)

Abbreviations: ART antiretroviral therapy; TB tuberculosis; HIV Human immunodeficiency virus.

*n = 6 with missing / unknown / other gender reported.

Participants were mostly between the ages of 20–25 years (65.8%), Black (91.2%), South African (73.7%) and female (54.8%). Just over 10% of the study participants were registered at a private university, and a third of students were covered by private health insurance. Males (44.6%) were mainly studying in the field of Science and Engineering, whereas most females (46.3%) were registered in Humanities and Education.

### Engagement in health services, including for HIV and TB

Half of all students (50.5%; 400/792) had visited a health facility in the last six months, and the most common reason for the visit were because they had been sick (n = 178/400; 44.5%) or for HIV testing (n = 116/400; 29.0%). Compared to females, males were less likely to visit the health facility in the last 6 months (42.6% vs. 57.4%; RR 0.74 95% CI 0.64–0.86). Health seeking behavior did not differ by private or government subsidized institution (29.5% vs. 49.6%; p = 0.061), but those from government subsidized institutions were more likely to visit the health facility for HIV testing compared to those from the private institution (22.0% vs. 15.6%; RR 1.40 95% CI 0.98–1.83).

Most students (48.9%; 387/792) indicated that they would first go to a public hospital or health care center to access health services while a third would rather go to a private doctor or private clinic (29.2%). Only 15.0% of the students reported that they would go to their campus clinic, 0.7% to a traditional healer and the remainder (6.2%) to other providers.

Just less than one-fifth of participants (17%) were not willing to comment on their HIV testing status. A third of the students enrolled in the study (30.6%) reported that they had never been tested for HIV. Among those respondents who had had an HIV test (n = 416; 52.5%), the majority (96.2%) had been tested in the past six months, and for a third (30.6%), this was the only test reported within the last five years. A majority (81.3%) of the students reported that they had not been screened for TB in the past six months. Among those that reported having an HIV test (n = 400) or being screened for TB in the past six months (n = 112), 92.3% (369/400) and 57.1% (64/112) had these tests after entering the tertiary education system.

### Perceived barriers to the uptake of health services

**Fig 1:**
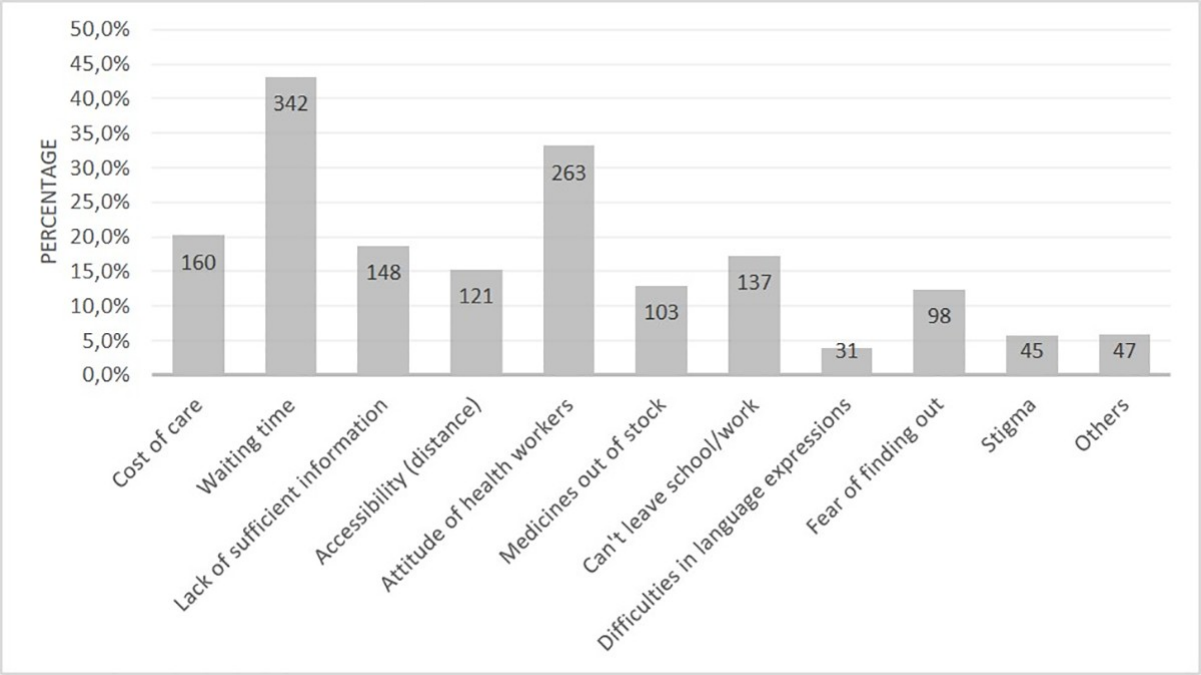
Perceived barriers to the uptake of health services as reported by first-year university students in Johannesburg, South Africa (n = 792). *multiple options apply.

The most common perceived individual and facility-related barriers to the uptake of health services in general as reported by students were long waiting time (n = 342, 43.2%), attitude of health workers (n = 263, 33.2%), cost of care (n = 160, 20.2%), lack of sufficient information (i.e., poor health literacy) (n = 148, 18.7%) and the inability to leave studies (miss classes or stay away from school) (n = 137, 17.3%) (Fig 1).

**Fig 2:**
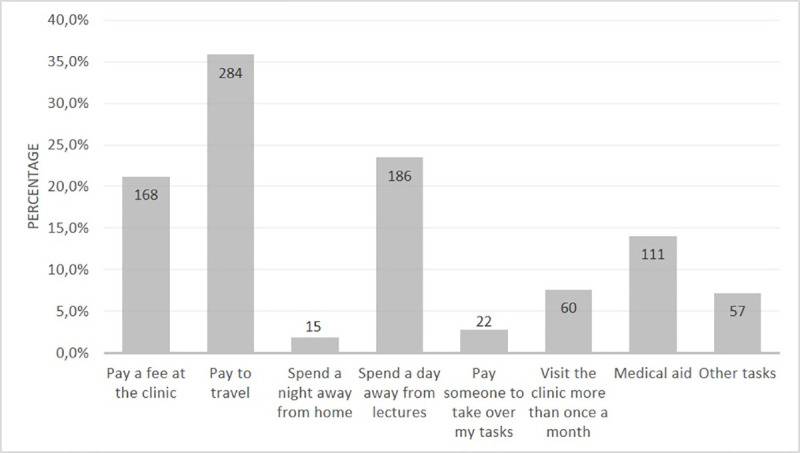
What first-year university students would need in order to visit the clinic/health care facility (n = 792). *multiple options apply.

Students reported what they would need to visit the clinic or health care facility. This included paying to travel (n = 284; 35.9%); missing a day of lectures (n = 186, 23.5%); paying a fee at the clinic (n = 168, 21.2%) and using medical aid/obtaining medical aid approval (n = 111, 14.0%) (Fig 2).

### Association between gender or recent engagement in HIV or TB services and perceived barriers to the uptake of health services

In general, there was no difference in the perception of barriers by gender with 56.8%, 15.1%, 8.5% and 12.2% of males (n = 352) and 52.1%, 19.4%, 8.3% and 16.6% of females (n = 434) reporting at least one, two, three and four or more barriers to the uptake of general health services (p = 0.823). Compared to males (n = 352), females were more likely to view attitudes of health workers (40.3% vs. 25.0%; RR 1.61 95% CI 1.25–2.08) and inability to leave/stay away from studies (20.5% vs. 13.4%; RR 1.54 95% CI 1.08–2.19) as potential barriers to accessing care for HIV and TB services (Table 2).

**Table 2:** Distribution of barriers to accessing care for HIV and TB services, stratified by gender.

	Gender			
Barrier	Male (n = 352)	Female (n = 434)	p-value	RR 95% CI
Cost of care	70 (19.9%)	89 (20.5%)	0.847	1.03 (0.75–1.41)
Waiting time	154 (43.8%)	185 (42.6%)	0.812	0.97 (0.79–1.21)
Lack of sufficient information	71 (20.2%)	77 (17.7%)	0.436	0.88 (0.64–1.21)
Accessibility (distance)	47 (13.4%)	74 (17.1%)	0.190	1.28 (0.89–1.84)
Attitudes of health workers	88 (25.0%)	175 (40.3%)	<0.001	**1.61 (1.25–2.08)**
Medicine out of stock	46 (13.1%)	57 (13.1%)	0.980	1.01 (0.68–1.48)
Inability to leave studies	47 (13.4%)	89 (20.5%)	0.017	**1.54 (1.08–2.19)**
Difficulties in language expression	16 (4.6%)	15 (3.5%)	0.446	0.76 (0.38–1.54)
Fear of finding out	47 (13.4%)	51 (11.8%)	0.528	0.88 (0.59–1.31)
Stigma	23 (6.5%)	22 (5.1%)	0.395	0.78 (0.43–1.39)

Abbreviations: RR Relative risk; CI confidence interval; bold p<0.05.

HIV testing was similar between males and females (52.3% vs. 53.0%), however, among those who had ever had an HIV test, males were less likely to be tested in the past 6 months (91.3% vs. 100%; RR 0.91 95% CI 0.87–0.95). More students amongst those who had been tested for HIV in the past 6 months (n = 400, 50.5%) perceived waiting time (47.8% vs. 40.6%; RR 1.17 95% CI 1.0–1.37) and attitude of health care workers (38.0% vs. 29.9%; RR 1.27 95% CI 1.06–1.55) as potential barriers compared to those who had not tested in the past 6 months.

When looking at TB screening in the past 6 months, a higher proportion of males reported being screened for TB compared to females (16.5% vs. 12.4%; RR 1.33 95% CI 0.95–1.88). Compared to students who had not been screened for TB in the past six months (n = 644; 81.3%), those who had been screened (n = 112,14%) were less likely to perceive medication stock out as a potential barrier (5.4% vs. 14.8%; RR 0.36 95% CI 0.16–0.81). Despite these barriers, 90% of students said they would seek care if they displayed any symptoms of TB.

### Sources of information about HIV and TB

**Fig 3:**
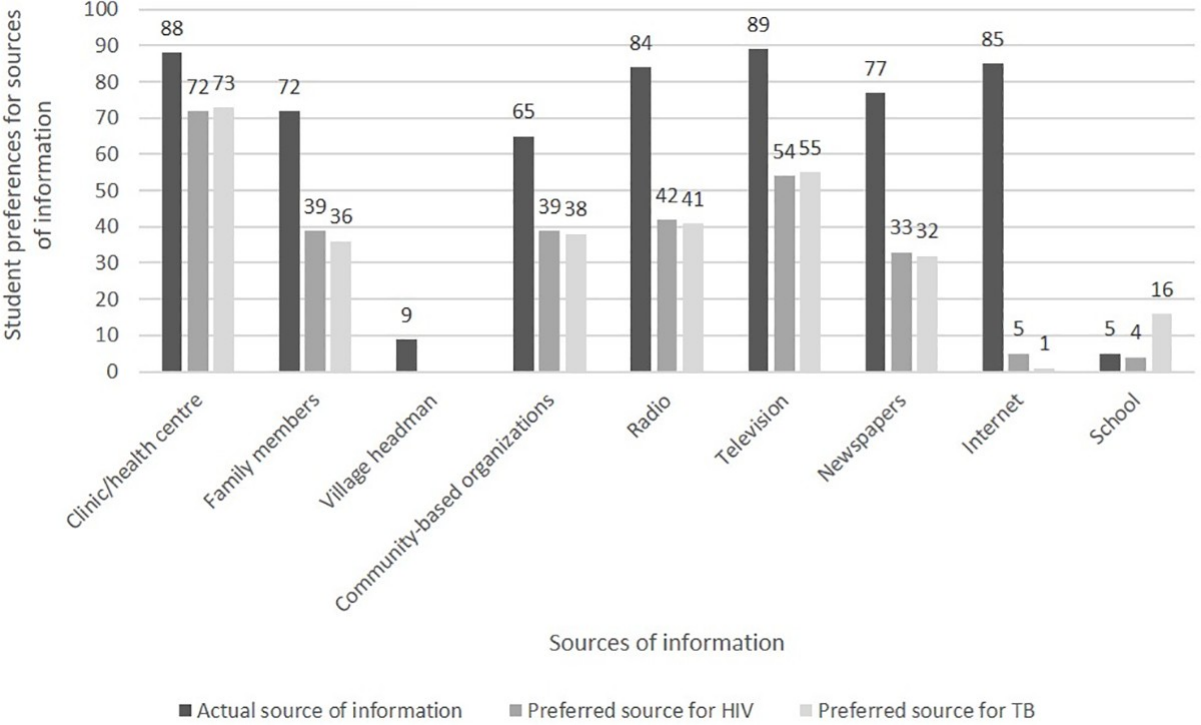
First-year university students reported their actual and preferred sources of information for HIV and TB (n = 792). *multiple options apply.

Students reported that they received most of the information they know about HIV and TB from a multitude of sources, including the television (89%), the clinic or health center (88%), internet (85%), and radio (84%). Very few (5%) students reported receiving information about HIV and TB from the university itself (Fig 3). The majority of the students (>70%) indicated that they would prefer to receive information about TB and HIV from a clinic or health center as opposed to other sources.

## Discussion

Our study of first-year university students found that in addition to commonly reported barriers (e.g., waiting times, poor staff attitudes, costs and stigma), lack of sufficient information (i.e., poor health literacy), and the inability to leave/stay away from studies were also important.

Many studies have documented the needs, barriers and gaps in providing comprehensive health services for young key populations, however these have mainly focused on primary care services or community-based settings [29–33]. University-based clinics offer a unique opportunity to deliver youth-friendly services tailored to meet the healthcare needs of young people [14]. However, the role of university health services in closing access gaps is not fully understood. To our knowledge, this is the largest, multi-site study in South Africa that has explored perceived barriers to the uptake of health services among first-year university students. In this study, we found that despite substantial access gaps, university services are not closing these gaps [34].

In our study, long waiting time was perceived by many students as a potential barrier, and most likely reflects public rather than campus health services that students are accessing. Potential solutions such as streamlining clinic flow/processes and improved provider-patient communication (e.g., use of signposts) to direct clients to correct queues and improve client satisfaction when there are staff shortages should be considered to reduce waiting time [35]. Campus health services could potentially address this barrier and, together with a model of appointment allocation, reduce waiting times and the negative impact of care-seeking on students’ studies [36].

University students perceived the inability to leave/stay away from studies as a potential barrier, with some students even reporting that they would need to spend a day away from lectures to access services. Health services become unavailable to students when clinic opening hours coincide with lecture hours, or when clinics are not open during weekends and public holidays. Other studies have shown that the inclusion of extended clinic hours, after-hours, and over weekends, in a comprehensive package of services improves uptake and linkage to care among adolescents [9, 12, 35, 37]. It is not clear why more females than males in our study perceived the inability to leave/stay away from studies as a potential barrier. It may be related to fear of losing access to quality education, fear of family reaction, family pressure (i.e., household chores, cost related to missing studies, time away from household or family responsibilities), parental consent requirements or perhaps related to stigma [38]. In other studies, stigma and discrimination have been identified as possible reasons why students prefer to access services from sources other than the university campus clinic [39].

Stigma can be a powerful force inhibiting the uptake of health services [16], but health facilities could overcome this by providing a welcoming and supportive environment so that clients feel comfortable to disclose their needs or problems [40]. In our study, while a small proportion (<10%) of students reported stigma as a perceived barrier to accessing services, responses to other questions (e.g., fear that others would find out or notice clinic attendance, distance to facility, attitudes of health worker and the fear of being judged by them) and the small number choosing to access campus health services (15.2%) suggest that anticipated stigma (i.e., the belief that prejudice, discrimination and stereotyping will be directed at the self from others in the future [41]) may be influencing behavior, and may do so differently among young adult males and females. In general, women are reported to experience anticipated stigma to a higher extent than men, while younger age is also related to higher levels of anticipated and internalized stigma [9]. Therefore, in addition to broadly focusing stigma-reducing interventions, special resources targeting young women may be warranted.

Females perceived staff attitudes as a potential barrier compared to males, and this could be because health care workers have negative attitudes toward providing reproductive health services to unmarried adolescents [42]. Other reasons such as fear that attendance at the clinic would be noticed by family, using medical aid/obtaining medical aid approval, requiring parental consent, feelings of embarrassment, and confidentiality concerns could mean that adolescents or young adults forgo accessing health services [43]. Since a third of the students in this study reported having private health insurance, most likely linked to their parents’ medical aid scheme, we speculate that for similar reasons, private health insurance could be perceived as a potential barrier in this population. Unfortunately, our study did not include a qualitative component to verify this. In our study, students perceived the cost of care as a barrier that would prevent them from accessing health care. In order to access services, some students would either have to pay a fee for the visit (e.g., consultation fee), pay to travel or use medical aid benefits. Transportation costs can be a significant barrier to health care access, especially for those who are unemployed or rely on financial support from their parents [44]. Because of their location, and depending on their fees structure, campus health services could better address these perceived barriers than public clinics by reducing the monetary and time costs associated with care-seeking [45].

We have previously shown that 55% and 52% of this student population have poor TB and poor HIV knowledge [8]. Of note, almost one in five students perceived lack of sufficient information as a potential barrier. In 2019, the Department of Basic Education recognized the decline in HIV prevention knowledge among learners and released new scripted lesson plans (SLP) to strengthen the teaching of Comprehensive Sexuality Education (CSE) in schools in South Africa. The CSE SLP's aim to address important topics systematically, thereby providing clear, non-judgmental information about sexual and reproductive health, HIV, and other STIs. The implementation of SLPS may help children and youth navigate their way through adolescence safely [46]. While very few students (<5%) identified language as a barrier, language is an important consideration when planning health promotion messaging. When asked about sources of information about HIV and TB, very few (5%) students reported receiving information from the university itself. This is a missed opportunity that campus health services could use to increase awareness and uptake of available services.

From our study, the most frequently reported perceived barriers to accessing services were related to time and cost. We also identified staff attitudes, stigma, or stigmatizing effects (e.g., attitude of health workers) as potential barriers to accessing services. In this study, we found that young females were more likely to perceive the attitude of health workers and the inability to leave school as potential barriers.

### Limitations

In addition to what is described in the original paper [8], the findings should be considered in light of the following study limitations. First, experience with health care services may be dependent of where students access services. We tried to ascertain this by asking students where they would access services but this response was mixed which made it difficult to accurately assess. Second, we included “fear of finding out” and “stigma” as potential barriers but did not ask specifically about TB or HIV related stigma which limited our ability to comment on the type of stigma; i.e., felt stigma (internal stigma or self-stigmatization), enacted stigma (external stigma, discrimination) or internalized stigma (e.g., experiences negative feelings or thoughts) [47]. Future work should include a validated stigma scale to access anticipated, enacted and internalized stigma. Lastly, our study included a structured questionnaire and did not include a qualitative component. In depth interviews with participants could have provided more depth, detail and understanding of the perceived barriers and appropriate solutions to overcome these.

## Conclusion

Few students favored campus health services over other health care providers, and utilization of campus providers was low. While just over half (50.5%) of the students in this study had accessed health services in the past 6 months, very few (15.0%) opted to use campus health services, and even less (5%) reported receiving information about HIV and TB from the university itself. This represents a missed opportunity to increase awareness through effective health promotion messaging and promote the uptake of available services among first-year university students who could benefit from these services for many years while registered at the university. In this study, we identified perceived barriers to accessing services among first-year university students and note how campus health services could address many of the barriers unique to university students, and close substantial access gaps in this population. To encourage students to utilize services, campus health facilities should also consider ways to reduce stigma and discrimination. Campus services should also recognize gender differences in care-seeking and use of health services and design services to meet the specific healthcare needs of young men and women.

## Supporting information

S1 Table Questions to first-year students to ask about perceived barriers, health seeking behavior and sources of information about TB and HIV. (DOCX)

## List of abbreviations

ART: antiretroviral therapy
HIV: human immunodeficiency virus
RR: relative risk
SES: socio-economic status
TB: tuberculosis
USA: United States of America
WHO: World Health Organization
