# Supplementary material for: Perceived barriers to the uptake of health services among first-year university students in Johannesburg, South Africa
Source: PLoS One. 2021 Jan 22;16(1):e0245427. doi: 10.1371/journal.pone.0245427 (PMC7822246; doi:10.1371/journal.pone.0245427)
Supplement: S1 Table — (DOCX) [file pone.0245427.s001.docx]

S1 Table. Questions to first-year students to ask about perceived barriers, health seeking behavior and sources of information about TB and HIV.

| **Question** | **Options** |
| --- | --- |
| 1. Where would you go to access services first? | - Public hospital - Public health center - Private clinic - Grocery store / vendor - Traditional healer - Spiritual healer - Private doctor (e.g. General Practitioner) - Mission hospital - Campus clinic - Other (specify) ____________ - Refuse to answer |
| 1. What would stop you from seeking health care for TB / HIV at your nearest health center? | (choose all that apply)   - Cost of care - Waiting time - Lack of sufficient information - Accessibility (distance) - Attitude of health workers - Medicines out of stock - Can’t leave/stay away from studies - Difficulties in language expressions - Fear of finding out - Stigma - Other (specify) ____________ |
| 1. To visit the clinic / health care facility, I would have to | (choose all that apply)   - Pay a fee at the clinic - Pay to travel - Spend a night away from home - Spend a day away from lectures - Pay someone to take over my tasks - Visit the clinic more than once a month - Other (specify) ____________ - Don’t know - Refuse to answer |
| 1. How would you get to a health care facility? | (choose all that apply)   - Walk - Commuter taxi - Cab - Bus - Other (specify) ____________ - Don’t know - Refuse to answer |
| 1. From what sources do you get information about TB / HIV? | (choose all that apply)   - Clinic/health center - Family members - Village headman - Community-based organizations - Radio - Television - Newspapers - Internet - Other (specify) ____________ |
| 6. Where would you prefer to get information about HIV from? | (choose all that apply)   - Clinic/health center - Family members - Village headman - Community-based organizations - Radio - Television - Newspapers - Internet - Other (specify) ____________ |
| 7. Where would you prefer to get information about TB from? | (choose all that apply)   - Clinic / health center - Family members - Village headman - Community-based organizations - Radio - Television - Newspapers - Internet - Other (specify) ____________ |
| 8. If you had a severe cough, night sweats or weight loss would you seek health services? | - Yes - No - Refuse to answer - Don’t know |
| 9. When was the last time you went to the health centre/clinic? | - Past week - More than a week but less than one month - More than 1 month but less than 6 months - More than 6 months but less than a year - More than a year ago - Never - Don’t know - Refuse to answer |
| 10. Why did you go to the clinic the last time? | - I took my child - I went for family planning - I went for antenatal care - I went because I was sick - I went for HIV testing - I went to pick up my medication - Others (specify) ________________ - Don’t know - Refuse to answer |
